
JOURNAL INFORMATION
==============================
NLM Title Abbreviation: Behav Neurol
Journal ID: Behav Neurol
NLM Journal ID: 8914585
Journal ID: BN

Behavioural Neurology

ISSN: 0953-4180
EISSN: 1875-8584
Publisher: Wiley

ARTICLE INFORMATION
==============================
PMCID: PMC5434327
PMID: 20543452
DOI: 10.3233/BEN-2009-0260
Article ID: 360562
Article version: 1
Subjects: Introduction

Vascular Cognitive Impairment

Gottesman Rebecca F.
Hillis Argye E.
Print publication date: 2010
Electronic publication date: 2010 Jun 10
Volume: 22
Issue: 1-2
First page: 1
Last page: 2
Received 2010 May 31; Accepted 2010 May 31
Copyright: Copyright © 2010 Hindawi Publishing Corporation and the authors.
Copyright year: 2010
License: This is an open access article distributed under the Creative Commons Attribution License, which permits unrestricted use, distribution, and reproduction in any medium, provided the original work is properly cited.
License URL: https://creativecommons.org/licenses/by/3.0/

==============================
Behavioural Neurology 22 (2009/2010) 1–2
DOI 10.3233/BEN-2009-0260
IOS Press

### Introduction

### Vascular Cognitive Impairment

Rebecca F. Gottesman and Argye E. Hillis

The role of systemic vascular disease in the development and manifestation of dementing illnesses is becoming increasingly important. Both epidemiologic and histopathologic studies have emphasized the role of vascular risk factors not only in the development of cognitive impairment and dementia, but also in the cognitive dysfunction that may result in the presence of both vascular and neurodegenerative disease. Because vascular risk factors can be easily identified, can be followed, and in theory, are treatable, management of vascular disease is one area of interest that might actually prevent cognitive impairment and dementia, although studies to date have not proven this in actual practice.

Although Alzheimer's disease (AD) is the most common type of dementia identified in a clinical setting, many individuals diagnosed with AD have cerebrovascular lesions identified at autopsy. Similarly, it is likely that what is diagnosed clinically as "vascular dementia" may also have a component of amyloid plaque deposition and neurofibrillary tangles. Because it is so difficult to differentiate between what is vascular disease and what is Alzheimer's disease, understanding the patterns of cognitive impairment that are seen in patients with primarily vascular dementias is one potential way of understanding the difference between the two.

This issue of *Behavioural Neurology* focuses on the associations between vascular disease and cognitive impairment. We have included papers using a range of methodologies, showing the importance of vascular disease in study of cognition. The manuscripts in this issue include both original research as well as reviews in the field of vascular cognitive impairment. In addition, we have included papers showing both the association between vascular disease in the acute setting of stroke and its effect on cognition, as well as the longer-term association between vascular disease and cognition.

In the acute assessment of cognition in individuals with cerebrovascular disease, Drs. Kim, Jeon, and Lee used a voxel based lesion-mapping analysis to determine whether syntactic comprehension is localized in similar regions in Korean patients as in native speakers of other languages, validating the utility of the Korean Syntactic Comprehension Test in the acute assessment of Korean patients with ischemic stroke. Also in acute stroke patients, Dr. Gottesman and her colleagues have evaluated the use of adding simple bedside neglect tests to the National Institutes of Health Stroke Scale, to determine if predicting of cerebral infarction size is improved. In a manuscript entitled "Rose-colored answers: Neuropsychological deficits and patient-reported outcomes after stroke", Dr. Barrett emphasizes the limitations of self-report in identifying neuropsychological deficits after stroke.

Drs. Mattioi and Vignolo describe a detailed assessment of an individual aphasic patient, followed over 10-months, in whom new non-phonologically based reading errors emerged, in the setting of improvement of reduction in other errors, consistent with the Summation Hypothesis. Dr. Cloutman and her colleagues also evaluate the pattern of reading performance in a series of 112 individuals with acute stroke.

In a paper entitled, "Dual task impairments in vascular dementia", Drs. Inasaridze, Foley, Logie, and Della Sala examine deficits associated with dual-task performance in individuals with vascular dementia, compared to normal controls. In their manuscript, Drs. Lamar, Price, Giovannetti, Swenson, and Libon explore the dysexecutive syndrome in individuals with ischemic cerebrovascular disease, using an empirical approach to neuropsychological assessment of individuals with cognitive impairment. In an original study of patients with coronary artery disease, Dr. Gottesman and col-

ISSN 0953-4180/09,10/$17.50 © 2009/2010 – IOS Press and the authors. All rights reserved leagues report an association between level of cardiac function and level of cognitive performance.

This collection of manuscripts addresses some of the many issues relevant to the study of patients with vascular cognitive impairment and vascular dementia. The field of vascular cognitive impairment is at the intersection of the study of cerebrovascular disease and the study of cognition, and collaboration between the two fields will be necessary for further progress in this area.

There are many issues we have not addressed in this issue. Future directions that we and others will investigate include the relationships between blood pressure, cardiac ejection fraction, anemia, and other factors that influence delivery of oxygen to the brain on cognitive function acutely and chronically, and the implications of these relationships for intervention, particularly in individuals with vascular disease. Others will investigate the relationship between APOE alleles, cholesterol receptors, and underlying pathological burden in vascular dementia and Alzheimer's disease, and how it relates to the degree of cognitive impairment. Another important issue is Cerebral Amyloid Angiopathy, which causes both vascular disease and dementia, and begs for effective treatment as well as prevention. New imaging techniques will play a large part in many of these investigations, as they have in many of the papers reported in this issue. New imaging of vasculature and the brain have been developed even during the writing of this issue, and will continue to be developed in the next decade.
